# Abundance and population characteristics of the invasive sea urchin *Diadema setosum* (Leske, 1778) in the south Aegean Sea (eastern Mediterranean)

**DOI:** 10.1186/s40709-021-00142-9

**Published:** 2021-05-21

**Authors:** Dimitris Vafidis, Chryssanthi Antoniadou, Konstantinos Voulgaris, Anastasios Varkoulis, Chrysoula Apostologamvrou

**Affiliations:** 1 School of Agricultural Sciences, Department of Ichthyology and Aquatic Environment, University of Thessaly, 38445 Nea Ionia, Magnesia Greece; 2 School of Biology, Department of Zoology, Aristotle University of Thessaloniki, 54124 Thessaloniki, Greece

**Keywords:** Density, Population structure, Biometry, Aegean sea, Invasive species, *Diadema*

## Abstract

**Background:**

The Indo-Pacific sea urchin *Diadema setosum* has invaded the Mediterranean Sea and has spread along many locations in the southeastern part of the basin, where established populations exist on the shallow subtidal rocky shore. *Diadema setosum* is a ubiquitous species, of particular ecological importance due to the high levels of grazing pressure it imposes on benthic communities. Its biology, however, is not adequately studied, especially along its introduced range of distribution. The present study examines the population status of *D. setosum* outside its native range, in the Dodecanese island complex, south Aegean Sea. Thirty-four stations located across 16 islands were surveyed by scientific SCUBA-diving (up to a depth of 10 m) in December 2019 and June-July 2020. Samplings included: (i) visual census along transects to estimate relative abundance and population density, and (ii) random collection of specimens from densely populated stations to assess biometry and reproductive condition (histological examination of gonads) of *D. setosum*.

**Results:**

*Diadema setosum* was found in 21 out of the 34 surveyed stations. The species had sparse populations of well-hidden individuals in rocky crevices, but with dense localized patches in Agathonisi, Leros, Kalymnos, Pserimos, Symi, Alimia and Chalki islands. In those seven islands, mean population density was 2.5 ± 1.48 individuals m^−2^. *Diadema setosum* had denser populations in shallower depths but larger dimensions in deeper; these results suggest segregated density and size patterns along a depth gradient. The size structure, according to the size frequency distribution of the test diameter, was unimodal with a fitted mode at 4.0–4.5 and 6.5–7.0 cm in shallow and deep populations, respectively. The examined morphometric relationships followed negative allometry, as previously suggested for the species within its native range of distribution, and test diameter appeared to be a good predictor of biomass. *Diadema setosum* specimens had immature gonads in winter and mature in summer, suggesting a synchronous reproductive pattern. These results conform to previous data from temperate populations of the species.

**Conclusions:**

Differences in local environmental conditions, e.g. hydrodynamics and habitat type, together with biotic interactions, e.g. recruitment and competition, probably shape *D. setosum* population in the south Aegean distributional range. The establishment of *D. setosum* has severe implications on benthic communities and local sea urchin populations demanding management measures to prevent the forecasted further expansion of this invasive species.

## Background

*Diadema* is a widespread and ecologically important genus of tropical sea urchins that contains nine extant species [1]. Among these species, *D. setosum* (Leske, 1778) has invaded into the Mediterranean basin in 2006 [2], and, currently, is among the established non-indigenous species (NIS) of the basin [3]. It is a sea urchin of Indo-Pacific origin; its native range extends from the mid Pacific to the East African coasts [4], including the Red Sea. It is especially abundant in the northern part of the Gulf of Suez [2]. Two separate clades of *D. setosum* have been recognized by molecular analyses, differing in their geographic distribution: clade A spreading throughout the Indo-West Pacific and clade B restricted around the Arabian Peninsula [4] and invaded the Mediterranean through the Suez Canal [5].

*Diadema setosum* inhabits the shallow sublittoral zone at depths ranging from one to 20 m, but most often the species aggregates around 4–6 m depth. It prefers rocky habitats and biogenic reefs, where it is hiding in crevices and under overhangs—especially during intense lighting—though, it can also be found on sandy bottoms and seagrass meadows [1]. The average size of the species is 6–7 cm and 3.5–4 cm in test diameter and height, respectively. The life span of *D. setosum* is around 3.5 years, with mature specimens weighting between 35 and 80 g [6]. The species exhibits variable reproductive patterns in different geographic areas, influenced by local environmental factors, such as temperature, lunar patterns and conspecifics and adults’ densities. *Diadema setosum* is an epibenthic grazer of particular ecological importance due to the high levels of grazing pressure it imposes on benthic communities. Under high densities, the species may transform rocky shores to barrens [1] and severely bioerode biogenic substrates, especially coral reefs [7]. Therefore, the population characteristics of this keystone sea urchin species may have profound cascade effects on the coastal ecosystem.

Recently, Muthiga and McClanahan [1] reviewed the biology of the genus *Diadema* covering several aspects, such as species evolution and biogeography, reproductive biology and recruitment, feeding ecology and grazing effects, growth and longevity, population dynamics, and community ecology and coexistence. This effort revealed that, as opposed to its congeners *D. antillarum* and *D. mexicanum*, the biology of *D. setosum* is poorly studied, and is mostly confined to tropical populations, despite being very common in many areas of its distribution. The few existing data from temperate populations are limited to the reproductive biology of the species [8–11], their grazing [12] and the bioerosive pressure they inflict in rocky biogenic habitats [13], and to the cytotoxicity of its bioactive compounds [14]. Focusing on the Mediterranean population, existing data refer exclusively to its occurrence [2, 15–26], whereas only a single study examined the genetic profile of the species and suggests further spread of *D. setosum* in the near future through larval transport [5].

Consequently, the present work aims to assess the current status of *D. setosum* in the Dodecanese complex (south Aegean Sea) by implementing a combination of non-destructive sampling techniques and random collection of specimens to gather information on density and biometry at spatial scales. Moreover, attempts to describe the reproductive status of the sea urchin in densely populated areas.

## Results

**Fig. 1:**
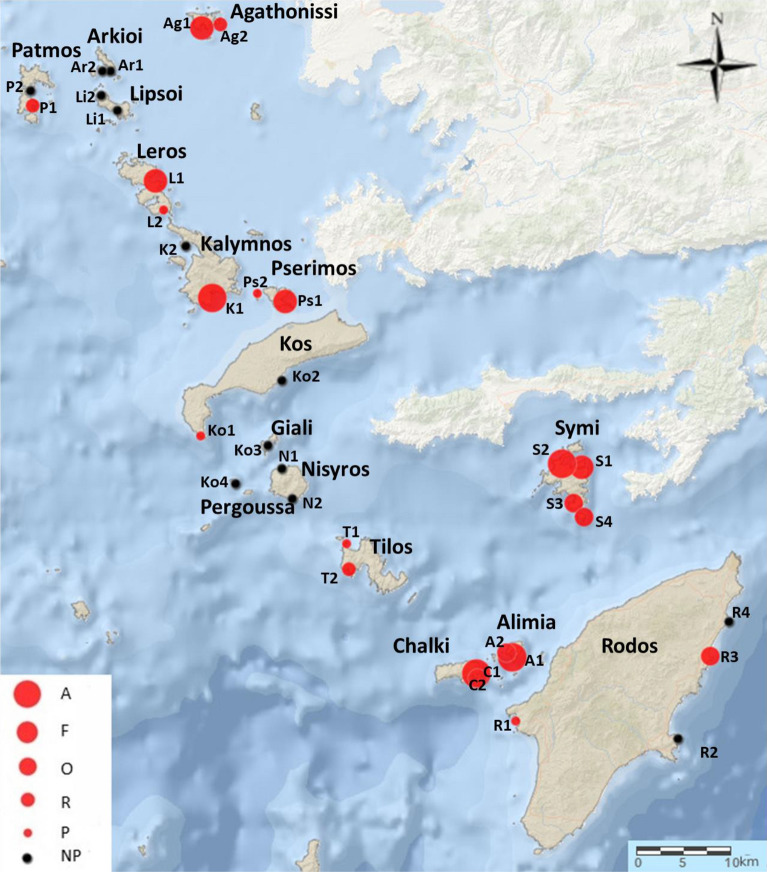
Sampling stations and relative abundance of *Diadema setosum* in the marine area of the Dodecanese island complex. *A* abundant, *F* frequent, *O* occasional, *R* rare, *P* present, *NP* no present

**Fig. 2:**
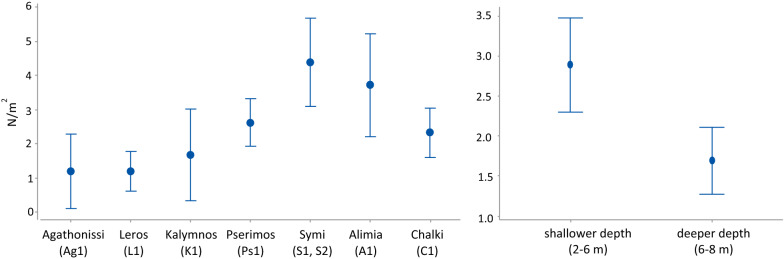
Mean population density ± Fisher LSD of *Diadema setosum* in the densely populated (A, C or F relative abundance grade) islands (left) and in the two depth zones (right) surveyed

*Diadema setosum* was recorded at 21 out of the 34 surveyed stations dispersed on 16 islands (Fig. 1). It settled mainly on rocky substrates along the shoreline, from 3 to 8 m depth, surrounded by boulders, detritic sediments and/or *Posidonia oceanica* meadows (Table 1). According to the applied ACFOR scale of relative abundance (see “Methods” section for numeric definition of relative abundance scale), *D. setosum* was present in five stations, had sparse populations in eight, and dense populations in another eight of the surveyed stations. This allowed a more accurate estimation of density in those latter eight stations, located in seven islands. Accordingly, the population density of *D. setosum* ranged from 0.8 to 5.3 individuals m^−2^ with an overall mean (x ± sd) of 2.50 ± 1.48 individuals m^−2^. Mean density showed significant variation among islands (F = 10.90, *p* < 0.01) and depth zones (F = 26.17, *p* < 0.01). Increased densities were recorded in the shallower depth zone, and in five stations (K1, S1, S2, A1, and C1, see Table 1), located in the islands of Kalymnos, Symi, Alimia and Chalki (Fig. 2).

**Table 1:** Location, code and geomorphological characteristics [depth, substrate type (R = rocky, B = boulders, D = detritic, M = meadows), slope (H = horizontal, MI = moderately inclined, I = inclined)] of the surveyed stations in the Dodecanese island complex (south Aegean Sea)

Island	Toponym	Latitude/Longitude	Code	Date	Depth (m)	Substrata Type/Slope	Method	Relative abundance	Density N m^−2^
Patmos	Grikou	37^o^18′02.3’’N 26^o^33′49.7’’E	P1	12/19	3–5	R/MI	T_500_	R	
	Meloi	37^o^19′47.2’’N 26^o^35′04.5’’E	P2	12/19	< 10	DM/H	T_500_	NP	
Agathonisi	A.Georgios	37^o^27′03.4’’N 26^o^59′15.1’’E	Ag1	12/19	3–6	R/MI	T_500_ / T_10_	F	1.2 ± 6.8
	A.Nikolaos	37^o^27′27.3’’N 26^o^59′39.1’’E	Ag2	12/19	3–6	R/MI	T_500_	R	
Arkioi	Tiganaki	37^o^22′01.8’’N 26^o^44′49.3’’E	Ar1	12/19	< 10	R/MI	T_500_	NP	
	Marathi	37^o^21′59.7’’N 26^o^43′38.0’’E	Ar2	12/19	< 10	RbM/MI	T_500_	NP	
Lipsoi	Moshato	37^o^19′17.8’’N 26^o^43′22.2’’E	Li1	12/19	< 10	RbM/MI	T_500_	NP	
	________	37^o^17′34.2’’N 26^o^45′47.8’’E	Li2	12/19	< 10	RbM/MI	T_500_	NP	
Leros	Agia Marina	37^o^10′31.4’’N 26^o^51′04.8’’E	L1	12/19	4 / 8	R/H – R/MI	T_500_ / T_10_	F	1.6 ± 8.3 / 0.8 ± 2.2
	Xirokampos	37^o^06′20.7’’N 26^o^52′21.2’’E	L2	12/19	4–6	RbM/MI	T_500_	P	
Kalymnos	Therma	36^o^56′15.5’’N 26^o^59′16.2’’E	K1	12/19	4–5	Rb/H	T_500_ / T_10_	A	2.3 ± 4.57
	Lepto	37^o^02′32.6’’N 26^o^55′36.1’’E	K2	12/19	< 10	Rb/H	T_500_	NP	
Pserimos	Vathi	36^o^55′52.6’’N 27^o^09′38.9’’E	Ps1	12/19	3–6	Rb/MI	T_500_ / T_10_	F	1.8 ± 8.46
	Plati	36^o^56′54.8’’N 27^o^05′32.7’’E	Ps2	12/19	3–5	Rb/MI	T_500_	P	
Kos	Charakas	36^o^40′34.7’’N 26^o^57′35.5’’E	Ko1	7/20	3–5	R/MI	T_500_	P	
	Kardamaina	36^o^48′43.4’’N 27^o^11′25.8’’E	Ko2	7/20	< 10	Rb/H	T_500_	NP	
Pergoussa	________	36^o^35′24.0’’N 27^o^02′36.4’’E	Ko3	7/20	< 10	RbM/MI	T_500_	NP	
Giali	________	36^o^40′01.2’’N 26^o^08′21.5’’E	Ko4	7/20	2–6	Rb/H	T_500_	NP	
Nisyros	Loutra	36^o^36′27.5’’N 26^o^09′03.8’’E	N1	7/20	< 10	Rb/H	T_500_	NP	
	Avlaki	36^o^33′26.7’’N 27^o^50′48.4’’E	N2	7/20	< 10	RbM/H	T_500_	NP	
Symi	Pedi	36^o^36′59.8’’N 27^o^51′34.1’’E	S1	7/20	4–6	R/I	T_500_ / T_10_	F	2.1 ± 3.9 / 1.3 ± 3.5
	Emporios	36^o^37′54.0’’N 27^o^48′30.8’’E	S2	7/20	4 / 8	R/MI	T_500_ / T_10_	A	4.5 ± 6.6 / 2.6 ± 5.1
	Panormitis	36^o^33′05.2’’N 27^o^50′44.1’’E	S3	7/20	4–6	Rb-D-M/H	T_500_	O	
	Seskli	36^o^32′43.9’’N 27^o^51′42.9’’E	S4	7/20	4–6	R/MI	T_500_	O	
Alimia	Tigani	36^o^15′19.6’’N 27^o^42′0.66’’E	A1	7/20	4–6	Rb-D-M/H	T_500_ / T_10_	A	4.4 ± 8.1
	________	36^o^15′30.6’’N 27^o^41′03.1’’E	A2	7/20	< 10	R-M/MI	T_500_	O	
Chalki	Limani	36^o^13′22.8’’N 27^o^36′47.2’’E	C1	7/20	2–4/6–8	R-B-M/H	T_500_ / T_10_	A	5.3 ± 5.9 / 2.1 ± 4.3
	Krevatia	36^o^12′52.7’’N 27^o^37′09.8’’E	C2	7/20	4–6	R-M/MI	T_500_	O	
Tilos	A.Antonios	36^o^28′11.2’’N 27^o^18′16.5’’E	T1	7/20	4–6	R-B-M/MI	T_500_	P	
	Limenari	36^o^25′24.5’’N 27^o^18′34.0’’E	T2	7/20	4–6	R-M/I	T_500_	R	
Rodos	Monolithos	36^o^08′51.9’’N 27^o^42′22.1’’E	R1	7/20	6	R-B-M/MI	T_500_	P	
	Lindos	36^o^05′32.4’’N 28^o^05′15.6’’E	R2	7/20	< 10	R-B/MI	T_500_	NP	
	Kolympia	36^o^14′52.6’’N 28^o^10′16.7’’E	R3	7/20	4–6	R-M/MI	T_500_	O	
	Ladiko	36^o^19′22.8’’N 28^o^12′43.0’’E	R4	7/20	< 10	R-B/MI	T_500_	NP	

Sampling date, method (T_500_ = 3 transect replicates each 1 × 500 m long, T_10_ = 4 transect replicates each 1 × 10 m long), relative abundance (A = abundant: > 40% SE, > 50 N per 100 m^2^; C = common: 20–40% SE, 10–50 N per 100 m^2^; F = frequent: 10–20% SE, 5–10 N per 100 m^2^; O = occasional: 5–10% SE, 1–5 N per 100 m^2^; R = rare 1–5% SE, < 1 N per 100 m^2^; P = present, NP = no present) and population density (mean N m^−2^ ± standard deviation, estimated in stations with A, C and F relative abundance grade) results of *Diadema setosum*. *SE* species’ expansion, *N* number of individuals

Overall, 160 individuals were measured to describe the size structure of the studied population: 120 from the shallower depth zone and only 40 from the deeper one. Their size ranged from 0.95 to 4.78 cm in height, and from 1.94 to 8.90 cm in diameter, with a mean (x ± sd) of 2.61 ± 1.04 cm (Ht), 5.03 ± 1.76 cm (Dt), respectively. Their biomass ranged from 3.77 to 248.95 g in weight with a mean (x ± sd) of 60.64 ± 48.74 g (tW). Mean size and weight showed significant spatial differences between the surveyed islands (ANOVA, see Table 2).

**Table 2:** ANOVA results of the spatial effects on biometry (Ht = test height, Dt = test diameter,) and biomass (tW = total weight) of the surveyed *Diadema setosum* population in the Dodecanese island complex (south Aegean Sea)

Source of variation		Ht		Dt		tW	
	df	F	*p*	F	*p*	F	*p*
Islands	6	11.88	0.0001	10.77	0.0001	14.92	0.0001
Depth	1	48.43	0.0001	67.14	0.0001	76.41	0.0001

**Fig. 3:**
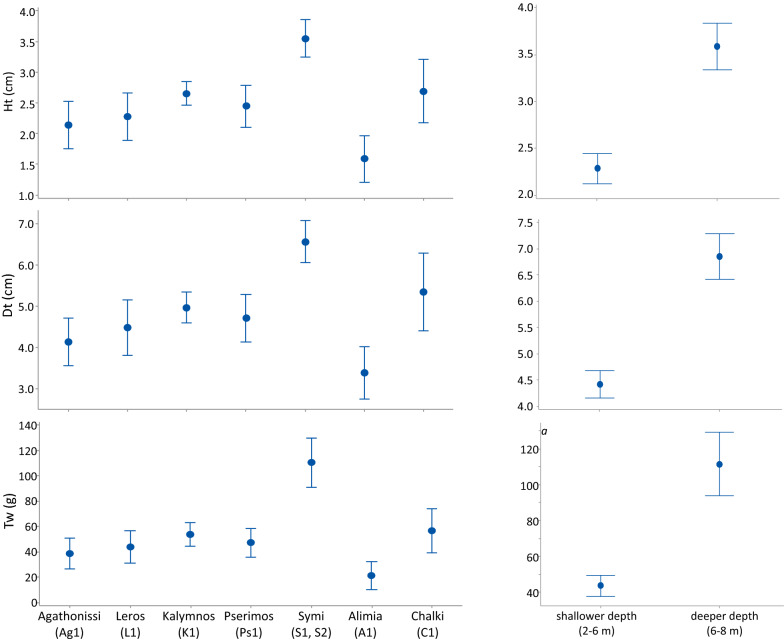
Mean size (Ht = test height, Dt = test diameter) and biomass (tW = total weight) ± Fisher LSD of *Diadema setosum* in the densely populated (A, C or F relative abundance grade) islands (left) and in the two depth zones (right) surveyed

**Fig. 4:**
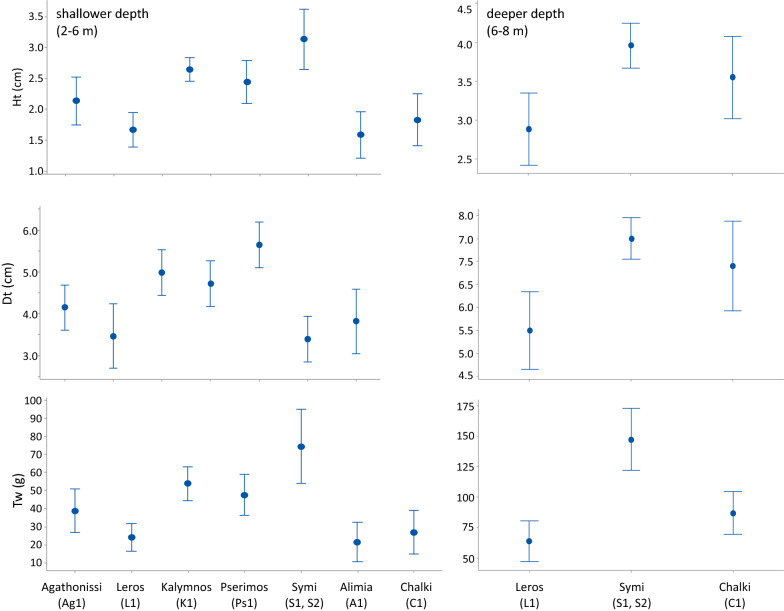
Mean size (Ht = test height, Dt = test diameter) and biomass (tW = total weight) ± Fisher LSD of *Diadema setosum* in the densely populated (A, C or F relative abundance grade) islands in the shallower (left) and lower (right) depth distribution of the species

Largest Dt, Ht and tW values were recorded in the stations of Symi and Chalki islands, intermediate in Agathonisi, Kalymnos, Leros and Pserimos, and decreased in Alimia (Fig. 3). Differences along the depth gradient were even more pronounce, with significantly increased values in deeper populations (Fig. 3). According to ANOVA and Fisher LSD post-hoc comparisons of *D. setosum* biometric features at each depth zone separately, the sea urchin had significantly (*p* < 0.001) larger dimensions (Dt, Ht) and weight (tW) in Symi, Pserimos and Kalymnos, and smaller in Alimia, Chalki and Leros in the shallow depth zone (Fig. 4, left graph). In the deeper depth zone, significantly larger urchins were measured (for Dt and Ht) in Symi and Chalki, and heavier urchins in Symi (ANOVA, *p* < 0.001; Fig. 4, right graph).

**Fig. 5:**
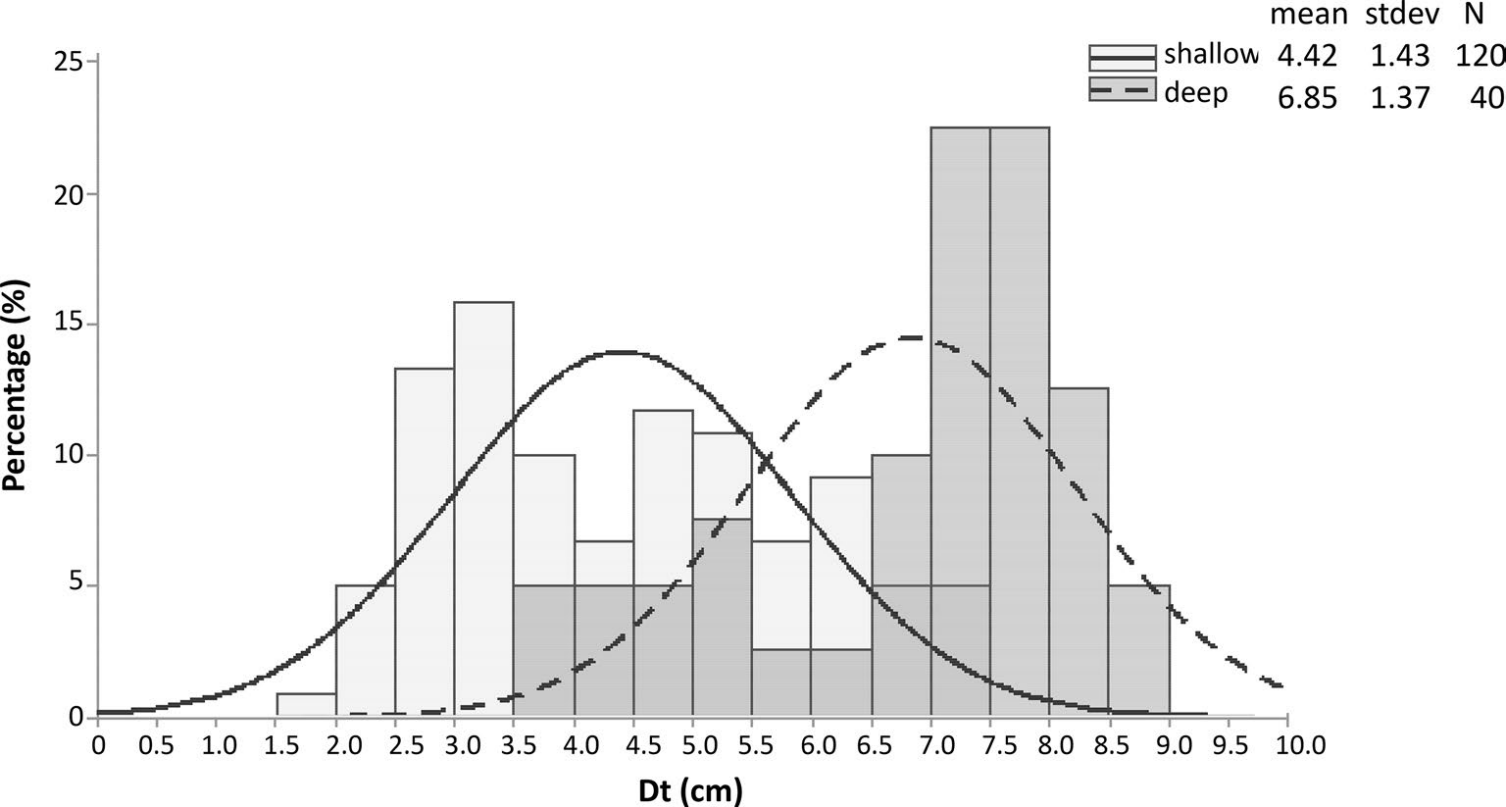
Size (Dt = test diameter)—frequency distribution of the studied *Diadema setosum* population per depth distribution zones surveyed (data were pooled over stations and islands)

**Fig. 6:**
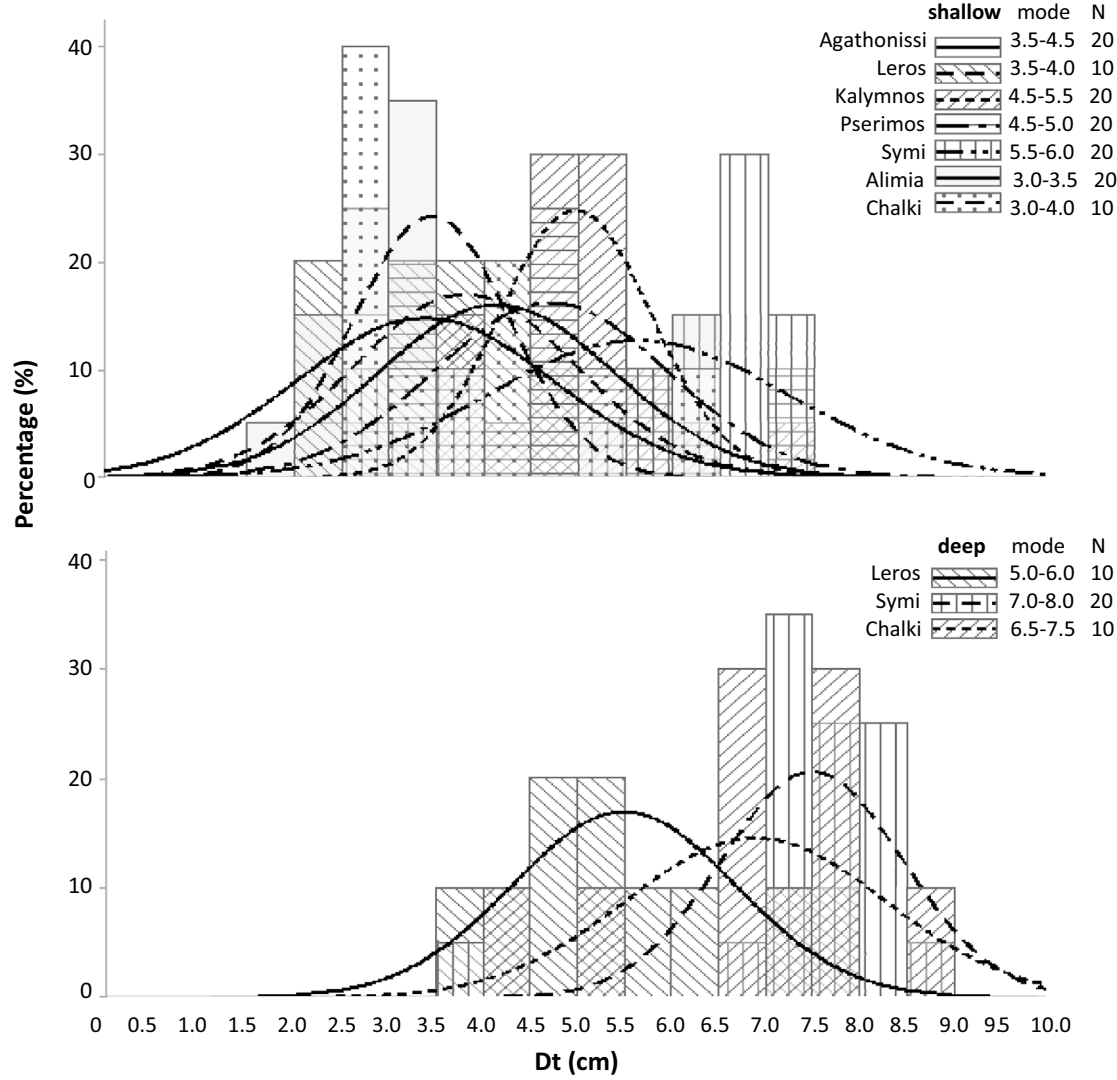
Size (Dt = test diameter)—frequency distribution of the studied *Diadema setosum* population per island, in the shallower (top graph) and deeper (lower graph) depth distributional zone of the species

Based on the above results, size frequency distributions (SFD) were calculated for each depth zone, and island separately. Overall, the studied populations were normally distributed with a fitted mode at 4.0–4.5 and 6.5–7.0 cm Dt in the shallow and deeper zones, respectively (Fig. 5). By focusing to the shallower depth zone, the lowest SFD mode was at 3.0–3.5 cm in Alimia and the largest at 5.5–6.0 cm in Symi (Fig. 6, top graph). A shift towards smaller urchins in the northern (Agathonisi, Leros) and southern islands (Alimia, Chalki) may be inferred from SFD analysis. In the deeper zone, a similar south to north pattern of decreasing size was also assumed, whereas the largest mode was detected in the easternmost population of Symi island (Fig. 6 lower graph).

**Fig. 7:**
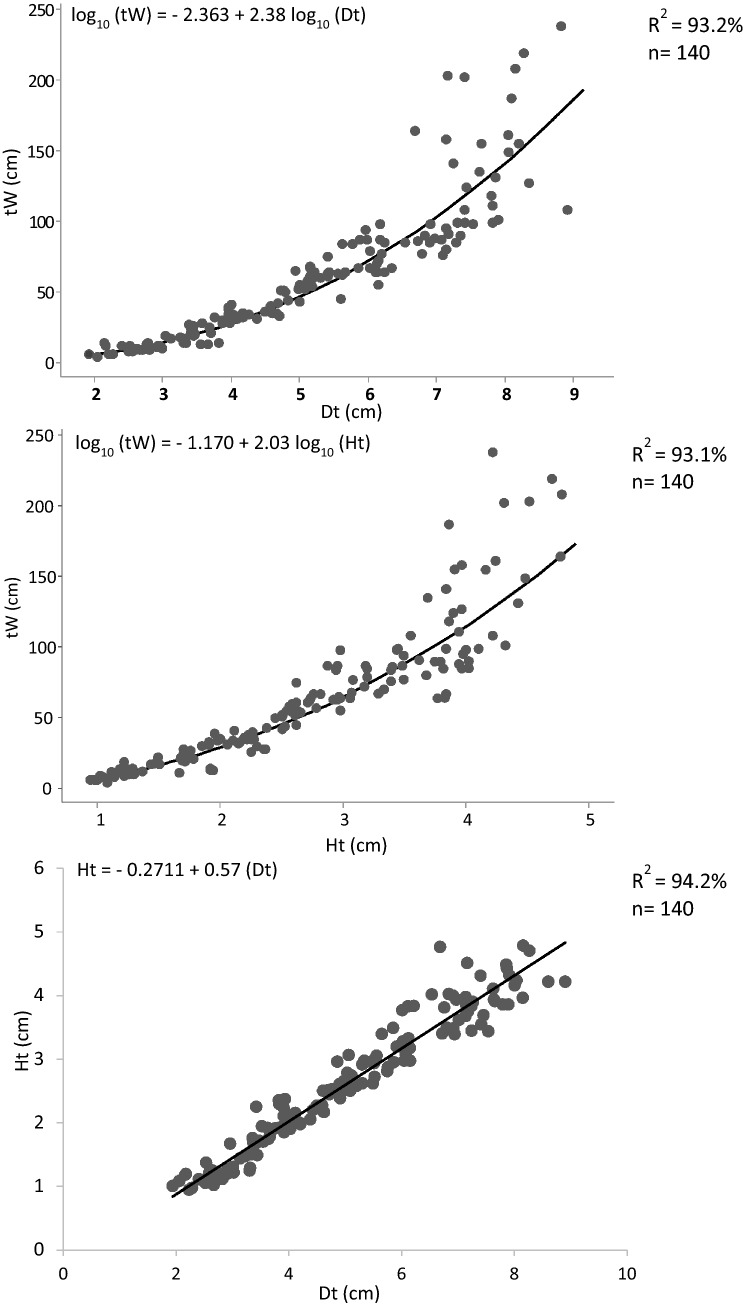
Morphometric relationships, weight/test diameter (tW/Dt), weight/test height (tW/Ht) and test height/test diameter (Ht/Dt) of the studied *Diadema setosum* population (data were pooled over islands and surveyed depths)

Biometric relationships tW/Dt, tW/Ht, Ht/Dt, estimated over the entire *D. setosum* population (Fig. 7) as well as per sampling depth (data not shown), were negatively correlated. The b coefficient was 2.38 for tW/Dt (t-test result under the null hypothesis of b = 3 at 95% confidence level: ts = − 6.45), 2.03 for tW/Ht (t-test results under the null hypothesis b = 3 at 95% confidence level: ts = − 7.52), and 0.57 for Ht/Dt (t-test results under the null hypothesis of b = 1 at 95% confidence level: ts = − 37.76). All three relationships had very high determination coefficient (> 90%) and thus, test diameter or height measurements proved to be efficient predictors of the urchin’s body mass.

**Fig. 8:**
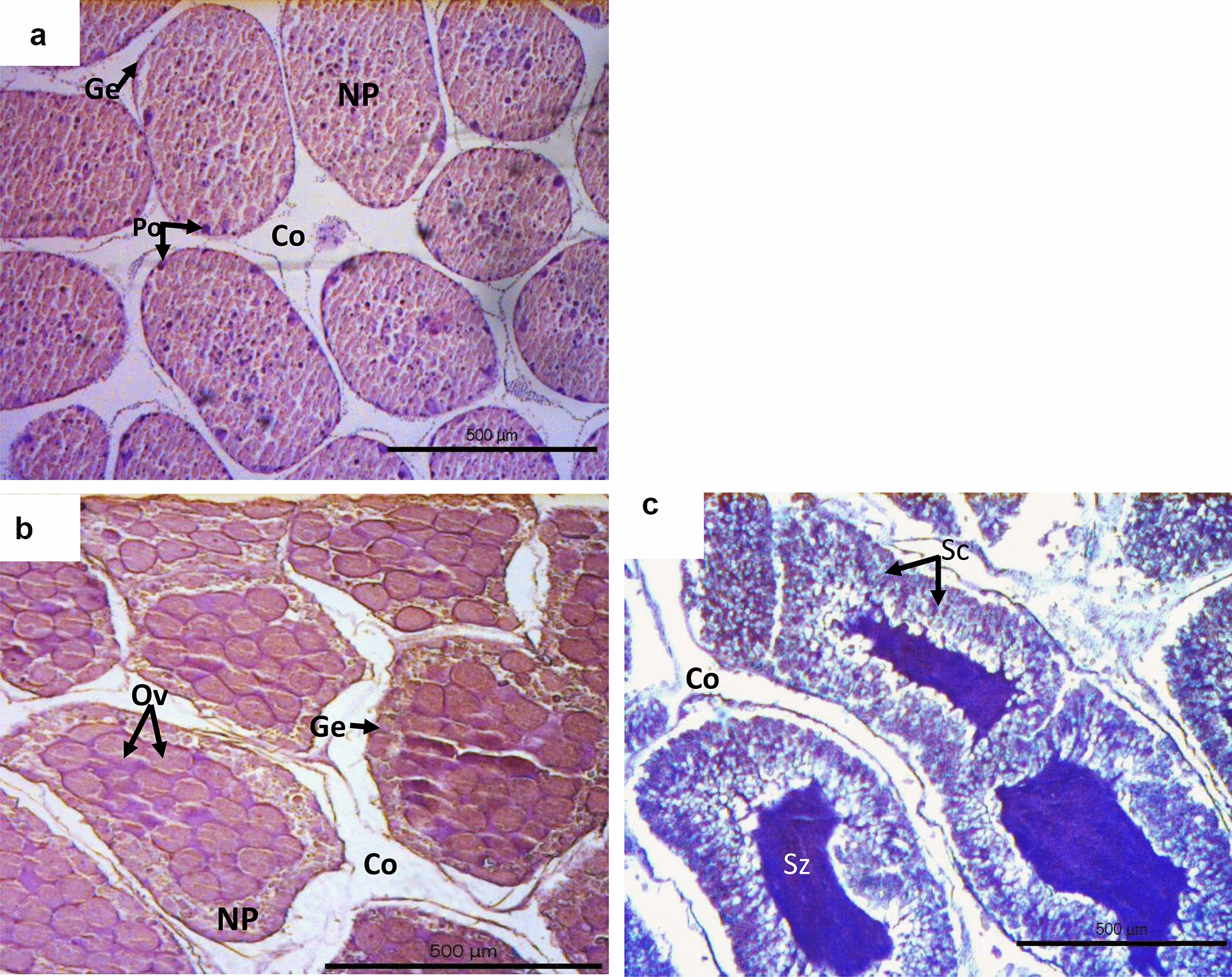
Reproductive stages according to Bronstein et al. [11] of the studied *Diadema setosum* population in Dodecanese island complex, observed in December 2019 and July 2020 samplings. **A** Stage II (recovering): clusters of previtellogenic oocytes formed in the ovarian germinal epithelium which may also occupy the central space of the female gametes. **B**, **C** Stage IV (mature): Ovaries (**B**) and testicular lumen (**C**) packed with mature ova and spermatozoa, respectively. Oocytes at different maturation stages are distinguished in the germinal epithelium. *Ge* germinal epithelium, *Co* coelom, *NP* nutritive phagocytes, *Po* previtellogenetic oocytes, *Ov* ova, *Sz* spermatozoa, *Sc* spermatocyte

Overall, 40 specimens were dissected to assess the reproductive status of the studied population; 20 from stations sampled in mid-December and another 20 from stations sampled late June to early July. Collection days were close to full moon in both seasons. In December, all sea urchins were in the recovering stage (Fig. 8A), whereas in June-July mature stages (Fig. 8B and 8C) prevailed in both male and female urchins (85% of the dissected specimens). In summer, immature urchins measured less than 3 cm in Dt from the shallow Alimia station (A1), whereas in December specimens ranged from 3.5 to 7.5 cm Dt.

## Discussion

It is difficult to evaluate the population status of *Diadema setosum* in the Mediterranean basin, after its successful invasion in 2006, as published data refer exclusively on the presence of the species (see Table 3) without any information on abundance, density or size structure. It is fairly evident, however, that the species has established populations in many locations along the Levantine and south, south-eastern Aegean Sea, and that *D. setosum* is further expanding towards the north Aegean and the Ionian Sea (see confirmed records in Table 3).

**Table 3:** Confirmed records of the NIS *Diadema setosum* in the Mediterranean (N = number of observed individuals, ND = no data provided)

Geographic area	Latitude/Longitude	Year	Depth	Substrata	N	References
Turkey						
Kas Peninsula	36^o^08′27’’N 09^o^39′18’’E	2006	15–18	Rocky	2	[2]
Antakya Bay	35^o^57′20’’N35^o^55′20’’E	2010	9	Rocky	3	[16]
Gokova Bay	36^o^55′11’’N 28^o^10′16’’E	2014	4	Rocky, sandy	2	[17]
Dardanelles	40^o^24′12’’N 26^o^45′43’’E	2018	4–6	Rocky	-	[23]
Greece						
Kastelorizo	36^o^09′10’’N 29^o^35′30’’E	2014, 2015	3–5	Rocky	5	[18]
Pserimos	36^o^56′36’’N 27^o^09′53’’E	2016	2/15	ND	2	[22]
Mandraki, Rhodos	36^o^26′57’’N 28^o^13′41’’E	2015	6	Rocky	1	[19]
Kolokytha, Crete	35^o^15′24’’N 25^o^44′31’’E	2016	5	Rocky	1	[20]
Proti Island	37^o^02′39’’N 21^o^33′32’’E	2020	6/15	ND	8/1	[26]
Kythira Island	36^o^09′31’’N 22^o^56′57’’E	2020	4/27	ND	2	[26]
Avlemonas, Kythira	36^o^13′31’’N 23^o^04′52’’E					[25]
Archangelos, Laconia	36^o^37′44’’N 22^o^52′35’’E	2019	2	Rocky	1	[25]
Pedi Bay, Symi	36^o^36′48″N 27^o^51′22″E	2018	0.5	Rocky	2	[24]
Gialos Bay, Symi	36^o^37′13″N 27^o^50′18″E	2019	0.5–2.5	Rocky	4	[24]
Cyprus						
Cyclop's Bay	34^o^04′46’’N 34^o^59′09’’E	2012, 2016	10–13	Rocky	2	[21]
Decosta Bay	35^o^00′27’’N 34^o^03′43’’E	2012	1/10	Sandy, rocky	2	[21]
Lebanon						
Monsef	34^o^10′59’’N 35^o^37′55’’E	2009	10–20	Rocky	1	[15]
		2019	2.5	Rocky	1	[25]
Israel						
Tel Aviv	32^o^05′01’’N 34^o^45′46’’E	2016	3.5–8	Rocky	1	[5]

*Diadema* species are pervasive, especially on shallow rocky and reef habitats [27]. A patchy distribution pattern of many *Diadema* species, including *D. setosum*, with hidden individuals and large local aggregations has been reported from many studied populations ([1] and references therein). In the currently surveyed marine area of the Dodecanese, *D. setosum* forms sparse populations of well-hidden individuals in rocky crevices, but with dense localized patches in specific sites, such as Symi, Alimia and Chalki islands. Although not fully understood, this pattern may be attributed to the prevailed flows of surface water currents affecting larval transport [28–30] or to gregarious settlement of young recruits and adults as an adaptive strategy to better defend against predators [1].

The oligotrophic nature of the study area, leading to low algal biomass productivity levels [31], creates feeding constraints for many invertebrates [32, 33]. This may be another factor against the establishment of dense *D. setosum* populations, except of specific areas, such as ports (Chalki island), aquaculture facilities (Symi island), thermal springs (Kalymnos island), and shelter bays (Alimia island), where algal coverage can support the trophic needs of this omnivorous sea urchin species [1, 8]. Direct competition with herbivorous fishes, such as the NIS *Siganus* spp. that thrives in the study area [34], may also have affected *D. setosum* densities. Unfortunately, there are no specific studies on intraspecific competition of herbivorous species or on their effects on algal coverage in the Dodecanese.

In the current study, several stations with dense patches of *D. setosum* have been detected mostly at the south-eastern part of Dodecanese, especially in Kalymnos, Symi, Chalki and Alimia; in those sites, the species density varied from 0.8 to 5.3 individuals m^−2^. Unfortunately, there are no other relevant data to compare the density of *D. setosum* within the Mediterranean Sea. By considering non-Mediterranean populations, where the species’ density ranges from 0.01 to 7.5 individuals m^−2^ in Kenyan reefs [1], from 2.2 to 6.05 individuals m^−2^ in Thailand reefs [35] and from 0.32 to 5.92 individuals m^−2^ in Hong Kong rocky reefs [13], the reported densities in the present study may be considered as moderate. Natural recruitment combined with overfishing of *D. setosum* primary predators have been proposed long ago to explain dense populations of the species [36]. However, recruitment studies on the congeneric *D. antillarum* showed great variability in spatiotemporal scales and were independent of adults’ density ([1] and references therein).

In the present study, the abundance of *D. setosum* decreased towards the deeper depth zone surveyed, i.e., below 5–6 m. The typical depth distribution of *D. setosum* usually ranges from the surface down to 10 m depth [37]. The studied sea urchin population constituted of much larger individuals in the deeper zone, as well. These results suggest a size-segregated pattern with depth, which might be explained by environmental differences, such as wave exposure, and rocky shore topography. Dislodgement risk by hydrodynamics induced by wave action has been assigned as the main factor driving the vertical distribution patterns of several sea urchin species, including those of the genus *Diadema* [38]. Specific data for *D. setosum* are missing, but according to relevant data for its congeneric *D. antillarum*, increased water movement practically restricts the distribution of the species at the shallowest strata of the rocky shores. The morphology of *Diadema* spp. is less adapted to resist water motion and its spines are extremely fragile and cannot support attachment to the substrate [38]. Accordingly, the species thrives in the deeper, low-flow, part of the reefs. In the surveyed wave-swept rocky shores, the shallower part (usually the first 2–3 m) was very steep, smooth and compact, without forming crevices, holes or other sub-horizontal structures that could offer refuge to *D. setosum*. Deeper down, the substrate was more heterogenous with many crevices and sub-horizontal formations constituting a much-preferred microhabitat for the species (authors’ personal observations).

According to the size-frequency distribution analysis, the studied *D. setosum* population is composed of medium and large sized individuals, in the shallower and deeper zones of its bathymetric distribution. Test diameter was an excellent predictor of the urchin’s biomass, whereas the growth of *D. setosum* followed negative allometry, as previously suggested for the species within its native range of distribution [39]. Test diameter or height increased at a relatively faster rate than its weight, and the same stands for two-dimensional growth (Ht/Dt) as well. This pattern may reflect the need for *D. setosum* specimens to quickly attain a large diameter to face predation, as small individuals may be more susceptible to fish predation [1]. *Diadema setosum* has a short initial growth rate in contrast to its congeners, though the reported rates vary between different populations [1]. This initial slow growth may explain the slow colonization rate of this ubiquitous species, as it took about five years to be detected again after its first report in Kas Peninsula. However, the size range of the studied specimens suggest the presence of mature and reproductive population in the Dodecanese area.

The reproduction of *D. setosum* varies greatly from one geographic location to another, with moonlight, tidal rhythms, age/size of urchins, and food availability among the factors influencing gametogenesis and spawning behavior [1]. The species reproduces throughout the year in the tropics, but with peaks at different times of the year [40]. In temperate populations, the species spawns in summer and seawater temperature is assumed to be the driving factor with values above 25 °C triggering gametogenesis [9, 11]. Though this study did not attempt to describe precisely the reproductive biology of *D. setosum*, it confirms the presence of mature specimens in all densely populated station during summer. In contrast, all examined individuals were at recovering stage in winter. These results conform to the reported breeding season from temperate areas.

## Conclusions

*Diadema setosum* has successfully invaded the Mediterranean basin, as well-established and flourish populations can be found in the Levantine basin and the south Aegean Sea. The species has sparse populations in the shallow rocky sublittoral zone (< 10 m) with locally dense patches of mature individuals in many islands of the Dodecanese. The studied population is probably shaped by a combination of environmental (habitat type, hydrodynamics) and biotic factors (recruitment, interspecific competition). As a keystone competitive superior [41] grazer, *D. setosum* may have a profound effect shaping benthic communities. In all densely populated surveyed stations, interestingly, it was the only sea urchin species found, as neither *Arbacia lixula* nor *Paracentrotus lividus*, the two most common regular sea urchins in the Aegean Sea and the Dodecanese [42–44], were observed. Further spread of *D. setosum* in the near future has been already implied [5] and is further supported by presented results. Accordingly, the implementation of a monitoring scheme to gather essential biological information together with efforts to manage and control the establishment of *D. setosum*—possible exploitation of its gonads [45] or bioactive compounds [14]—and prevent further expansion of this invasive species are urgently needed.

## Methods

### Study area

The study was carried out at the Dodecanese island complex, located in the south Aegean Sea. In the marine area of the Dodecanese, water masses are warm (16–27 °C), saline (around 39–40 psu) and oligotrophic [32, 33]. One to four stations were selected at random on each of the sixteen surveyed islands (Fig. 1); most of them are continental in geologic origin, whereas Nisyros and Gialy are of volcanic origin [46]. Samplings were made in December 2019, and in June & July 2020, by scientific SCUBA diving in the shallow sublittoral zone, i.e., up to 10 m depth. They included a combination of visual census and random collection of *D. setosum* specimens to assess abundance and basic characteristics of the species’ population. At all stations, the sea bottom consisted of rocky substrates mixed with patches of sandy detritic sediments and interspersed *Posidonia oceanica* beds. The main geomorphological features of sampling stations are given in Table 1.

### Abundance and biometry

The semi-quantitative ACFOR scale of relative abundance [47, 48] was applied to broadly estimate the spatial patterns of the species density, by diving along three replicate transects 500 m, each. The ACFOR scale has five categories, modified as follows to better fit the size of the studied species. A = abundant, a species found almost everywhere, expanding to over 40% of the surveyed area, and/or with over 50 individuals per 100 m^2^. C = common, a species found almost everywhere but not as dominant as in A, expanding from 20 to 40% of the surveyed area, and/or with 10 to 50 individuals per 100 m^2^. F = frequent, a species found in many places, expanding from 10 to 20% of the surveyed area, and/or with 5 to 10 individuals per 100 m^2^. O = occasional, a species found in few places, expanding to 5–10% of the surveyed area, and/or with 1 to 5 individuals per 100 m^2^. R = rare, a species found in one or two places, expanding to 1–5% of the surveyed area and with less than 1 individual per 100 m^2^. When a species is found in the surveyed area but with meaningful abundance is assessed as present (P), while when being apparently absent from the surveyed area as no present (NP). Concurrently, seawater temperature, salinity, pH, and dissolved oxygen were recorded with an autographic conductivity-temperature-depth sensor, CTD (SeaBird Electronics, Washington USA).

At the stations having F or higher abundance grade, a more precise estimation of the species abundance was made. In those cases, population density was directly estimated using belt transect sampling [33, 49]. Thus, four replicate transects 1 × 10 m—covering 10 m^2^ each—were conducted at each station. In stations where the sea urchins expanded over a broad depth range, the replicate transects were equally dispersed at the shallower, i.e., 2–4 m, and the deeper, i.e., 6–8 m, depths, of these zones; transects were parallel to each depth contour. Along each transect, all living *D. setosum* individuals were counted, and five sea urchin specimens were randomly collected to estimate the size structure of the studied population. The fresh sea urchin specimens were measured on board for test diameter (Dt) and height (Ht), at ambitus avoiding spines, using an electronic caliper (Mitutoyo Corporation, Takatsu Ward, Japan, 0.01 mm precision), and drained for 5-min on filter paper. Each specimen was, then, weighted for total weight (tW) using an electronic scale (0.01 g precision). Overall, the sample size for biometry was 160 *D. setosum* individuals.

Analysis of variance was applied to examine differences in population density of *D. setosum* between islands and depths (both treated as fixed factors) using the general linear model [50]. The same analysis was applied to examine relevant spatial differences in the estimated biometric variables (Dt, Ht, tW) of the sea urchin. Prior to the analyses, data were tested for normality with the Anderson—Darling test, while the homogeneity of variances was tested with Cohran’s test and, when necessary, data were log-transformed. The Fisher LSD test was used for post hoc comparisons. ANOVAs were performed using the SPSS software package (IBM SPSS statistics v.25, IBM Corp, Armonk, New York, USA).

Size frequency distributions were constructed per 0.5 cm size class increments using Dt data [35], and the modal length was identified by fitting a normal distribution curve [51].

Morphometric relationships, i.e., height/diameter, weight/diameter and weight/height, were estimated using the linear (Ht = a + bDt) or the power function (tW = aDt^b^ which equals to LogtW = loga + bLogDt and tW = aHt^b^ which equals to LogtW = loga + bLogHt) and applying a regression analysis. The association degree between variables was calculated by the determination coefficient (R^2^), while a t-test with a confidence level of 95% was applied to detect whether the relative growth rates of the urchins’ biometric characters were isometric (Ho: b = 1 for Ht/Dt or b = 3 for tW/Dt and tW/Ht) or allometric (H1: b ≠ 1 for Ht/Dt or b ≠ 3 for tW/Dt and tW/Ht).

### Histology

Five of the collected (collection days: 11–12/12/2019 and 3–6/7/2020 were close to full moon: 12/12/2019, 5/7/2020, in both seasons) sea urchin specimens for biometry at each station were dissected to remove the five gonads, which were immediately fixed in 10% neutral buffered formalin solution. Fixed gonads were further processed in the laboratory to assess the reproductive status of the sea urchins using histological examination [49, 52]. The middle portions of each specimen gonadal tissues were placed in cassettes and inputted in histokinette (Leica TP 1020, Leica Microsystems GmbH, Nussloch, Germany) for dehydration (immersion in ethanol solution of increasing concentrations), clearing (immersion in xylene solutions to replace ethanol with an organic dissolvent), and embedding in liquid paraffin wax. The gonadal tissue paraffin blocks were left for cooling (Leica EG 1150H Leica Microsystems GmbH, Nussloch, Germany); then, the mold was removed and the blocks were mounted on a microtome (Slee Mainz Cut 5062, SLEE medical GmbH, Mainz, Germany) for sectioning (5 µm sections). The sections were stained with the hematoxylin–eosin regressive staining procedure [49, 52], covered with Canada balsam mounting medium, and observed under light microscopy connected with a digital camera (ProgRes Plus 2.1, JENOPTIC Optical Systems GmbH, Jena, Germany). The histological sections were photographed in appropriate magnification scale using the software Progress Capture 2.1. The different developmental stages of gametogenesis were assessed according to Bronstein et al. (2016) [11].

## Data Availability

The datasets used and/or analyzed during the current study are available from the corresponding author on reasonable request.

## Abbreviations

NIS: Non indigenous species
ACFOR scale: A = abundant, C = common, F = frequent, O = occasional, R = rare relative abundance scale
Dt: Test diameter
Ht: Test height
tW: Total weight
SFD: Size frequency distribution
